# Bistable, Irregular Firing and Population Oscillations in a Modular Attractor Memory Network

**DOI:** 10.1371/journal.pcbi.1000803

**Published:** 2010-06-03

**Authors:** Mikael Lundqvist, Albert Compte, Anders Lansner

**Affiliations:** 1 School of Computer Science and Communication, Department of Computational Biology, Royal Institute of Technology (KTH), Stockholm, Sweden; 2 Institut d'Investigacions Biomèdiques August Pi i Sunyer (IDIBAPS), Barcelona, Spain; 3 Department of Numerical Analysis and Computer Science, Stockholm University, Stockholm, Sweden; RIKEN Brain Science Institute, Japan

## Abstract

Attractor neural networks are thought to underlie working memory functions in the cerebral cortex. Several such models have been proposed that successfully reproduce firing properties of neurons recorded from monkeys performing working memory tasks. However, the regular temporal structure of spike trains in these models is often incompatible with experimental data. Here, we show that the *in vivo* observations of bistable activity with irregular firing at the single cell level can be achieved in a large-scale network model with a modular structure in terms of several connected hypercolumns. Despite high irregularity of individual spike trains, the model shows population oscillations in the beta and gamma band in ground and active states, respectively. Irregular firing typically emerges in a high-conductance regime of balanced excitation and inhibition. Population oscillations can produce such a regime, but in previous models only a non-coding ground state was oscillatory. Due to the modular structure of our network, the oscillatory and irregular firing was maintained also in the active state without fine-tuning. Our model provides a novel mechanistic view of how irregular firing emerges in cortical populations as they go from beta to gamma oscillations during memory retrieval.

## Author Summary

The basic computational principles of the brain are still unknown, and one major reason for this is related to the difficulties in simultaneously measuring detailed data from a sufficiently large number of cells. In techniques where populations of cells are monitored, resolution is low. Computational models have no such measurement limitations and can be constrained by several experiments at different levels of granularity, enabling testing of the biological plausibility of different computational theories. One such theory, the attractor network paradigm, has gained increasing support over the past twenty years by, for instance, comparing the output of attractor memory models to population data and spike frequency modulations of neocortical neurons. We take this comparison further by also looking at the fine-structure of activity in a network model with a novel modular structure also seen *in vivo*. This allows the network to operate in a new dynamic regime. In particular, we reproduce the irregular low-rate spiking of single cells *in vivo*, which has previously been a challenge for attractor network models. Oscillations in field potentials at gamma and beta frequencies, again believed to be connected to, or even essential for, attention and consciousness, emerge as a feature of the underlying dynamics of the model.

## Introduction

Persistent activity in prefrontal and parietal neurons has been identified as a neural correlate of working memory. Indeed, neurons in these areas show elevated firing for specific memoranda during the delay period of a working memory task [1]–[3]. Recent studies have analyzed the temporal structure of these neuronal spike trains, and its modulation during the task [4]–[7]. The data indicates that spike trains are highly variable across all task epochs [5], [7], while local field potential recordings suggest that the underlying neural populations present gamma-range oscillations [4]. These experimental findings (selective persistent firing, irregular spike trains, and population oscillations) pose important constraints on mechanistic models of working memory in the cortex.

Attractor network models have replicated and permitted the analysis of bistability of firing rates [8]–[11] i.e. the coexistence of a non-selective *ground state* and several possible active states, where one population shows an elevated-rate *foreground* activity and the rest a low-rate *background* activity. Highly irregular spike output in the ground state has been achieved by means of balance between excitation and inhibition [8], with spiking driven by fluctuations in the input [12], [13]. But the balance is typically lost when the network enters an active state, and persistent activity is typically more regular than background activity. Recently, some modeling efforts have succeeded in achieving highly variable foreground firing [14]–[16].

Here we investigate a model that operates in a new regime, with low-rate firing and population oscillations in both the ground and the active state (referred to as bistable oscillatory regime). This has several advantages, such as 1) a very robust bistability that does not require fine-tuning as in previous models; 2) it reproduces oscillations during delay activity which are prominent *in vivo* [4]; 3) there is no rate dependence on variability in contrast to existing models; 4) firing rates are low and there is only a small gap in firing rates between ground state and foreground activity in agreement with experimental results [5].

To investigate this we used and modified a previously developed spiking attractor network model, which demonstrated perceptual and memory operations such as memory recall, spontaneous attractor wandering and attentional blink [17]. We analyzed the statistics of neuronal activity and found a good match with experimental data during visual working memory tasks [4]–[7].

We used the same model neurons as in our previous investigations. However, the phenomena studied here are likely not dependent on details of the model neurons since preliminary results from two of our recently developed models with the same network architecture but comprising Hodgkin-Huxley type point-neurons and integrate-and-fire neurons respectively show much the same dynamics. A comparison of models employing different types of model neurons as well as a more in depth theoretical analysis of the phenomena reported here is certainly desirable but outside the scope of the current paper.

Oscillatory activity is compatible with attractor network models of working memory [18], [19], provided a depolarizing mechanism with long-time constant supports network dynamics, such as NMDAR-mediated synaptic currents [20] or slow intrinsic depolarizing currents [19]. We have found that a modular network structure in terms of hypercolumns stabilizes oscillatory activity in ground and active states, even in the absence of such slow depolarizing currents. High variability can emerge in such an oscillating network [21]. Hypercolumnar modularization therefore provides spike-train variability and bistability without fine-tuning.

## Results

**Figure 1:**
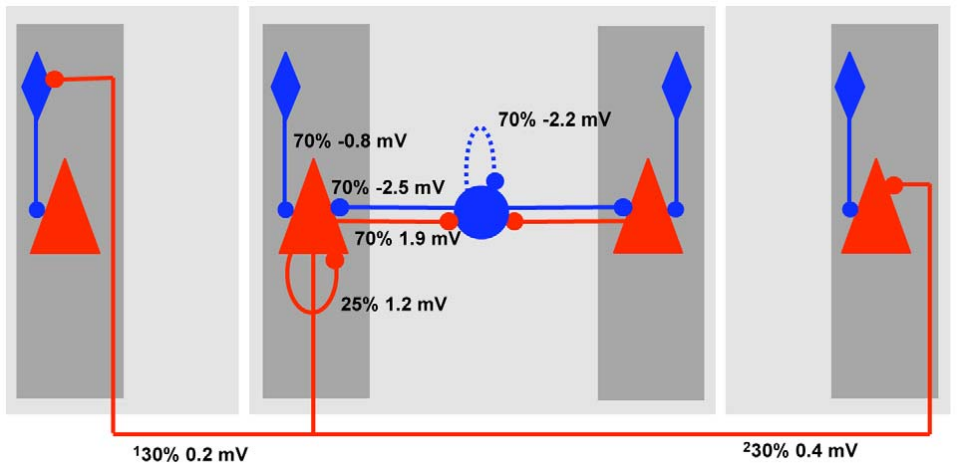
Schematic wiring diagram of the network model, with connectivity densities and average post-synaptic potential amplitudes as measured in the soma indicated. Hypercolumns are shown with light grey background, minicolumns with dark grey. The middle hypercolumn shows the mutual inhibition via basket cells between minicolumns in the same hypercolumn. The pyramidal cell to the left in this column shows how pyramidal cells project locally and globally. Percentages are given as the chance of one cell of the pre-population being coupled to one cell of the post-population. Note that global connectivity is exaggerated since the number of hypercolumns is down-scaled. Each cell sees about the same number of active synapses as it would in vivo assuming 1% activity. ^1^Connectivity of pyramid-RSNP cells given the two minicolumns are in different patterns, otherwise 0%. ^2^Global connectivity of pyramid-pyramid given the two minicolumns are in the same pattern, otherwise 0%.

We studied a computational network model of a neocortical layer 2/3 circuit with a minicolumnar and hypercolumnar structure, and a diversity of interneuron classes (see Methods). In brief, a cortical minicolumn was composed of thirty pyramidal cells [22], [23], one soma targeting basket cell and two dendrite targeting regular-spiking non-pyramidal (RSNP) interneurons (possibly double bouquet cells) of equal selectivity [24]–[26]. Fourty-nine such minicolumns of distinct selectivity were coupled to each other mainly through mutual inhibition to form a hypercolumn. The full model consisted of nine hypercolumns, which were mutually connected through excitatory conductance-based synapses. These excitatory projections across different hypercolumns targeted pyramidal neurons in minicolumns sharing the selectivity of presynaptic neurons and RSNP neurons in minicolumns of dissimilar selectivity to that of presynaptic neurons (Figure 1). This specific organization of connectivity among a diversity of cell classes is consistent with the known physiology and anatomy of the neocortex [25], [27]–[29] but its implications for persistent activity in working memory are still unknown. We describe in the following how this architecture instantiates novel mechanisms for persistent activity in our cortical network model.

### Bistable, low rate and irregular firing

**Figure 2:**
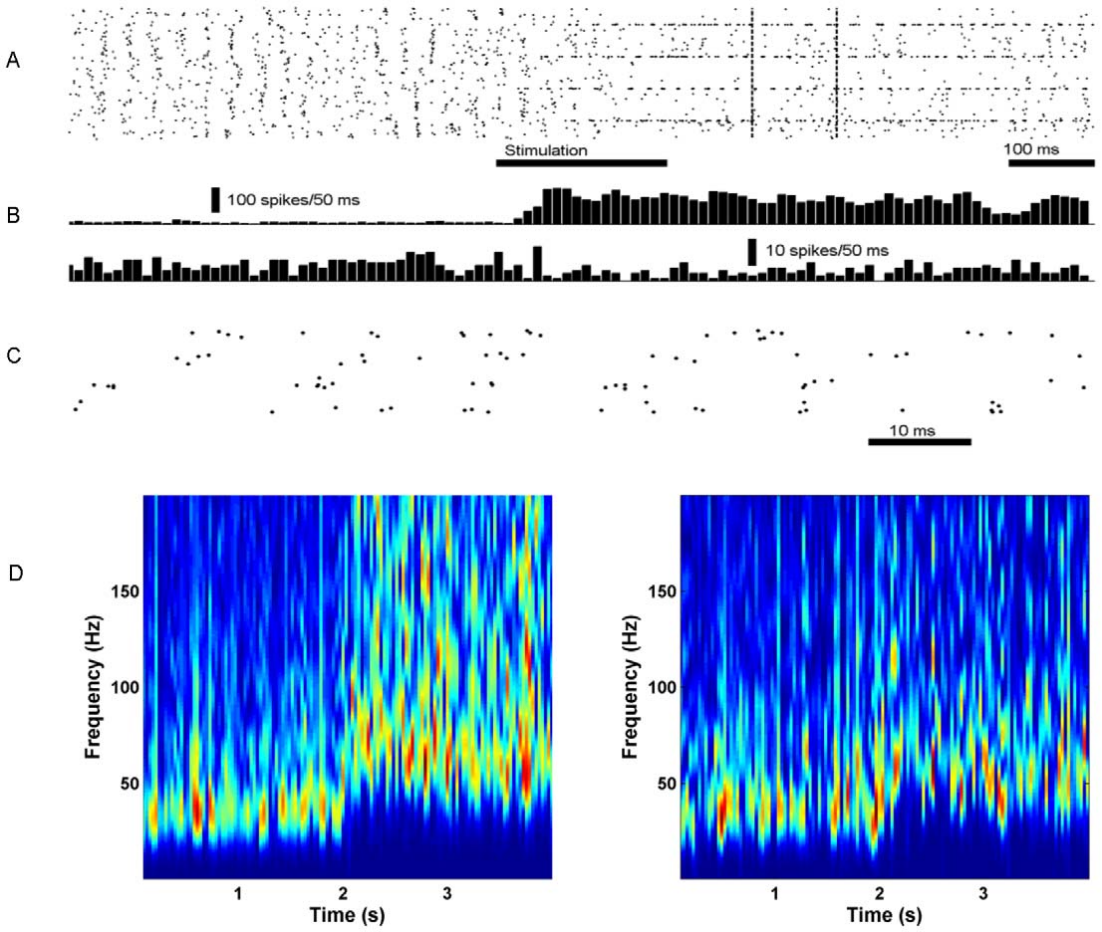
Spike raster showing bistability. A: A subsample of 5880 pyramidal cells (4 out of 9 hypercolumns) is shown. Each dot represents a spike occurring at a particular time (*x*-axis) and in a particular cell (*y*-axis). In the beginning of the simulation the stable, non-specific ground state was active. When a part of a pattern (first minicolumn in 5 out of 9 hypercolumns, see Methods) was stimulated it completed and was then persistently active, even after stimulation terminated. The foreground pattern consisted of the first minicolumn in each hypercolumn, so the activity after stimulation also marks the borders between the hypercolumns. Each of the four highly active and synchronous bands is the collective spike output of 30 pyramidal cells within the first minicolumn. The three bottom hypercolumns in the raster plot received direct stimulation and activated the top hypercolumn. After stimulation, the background pyramidal cells lowered their firing rates. B: Activity histograms of 30 pyramidal cells (top) going from ground state to foreground in the active state, and 30 pyramidal cells (bottom) going from ground state to background. The vertical bar marks 10 *s^−1^* (top) and 1 *s^−1^* (bottom), respectively, measured as the number of spikes divided by time and number of cells. C: Zoom in of the part of the spike raster that is indicated by the dashed vertical lines in A. Only the cells in the foreground population are shown. Active minicolumns are not tightly synchronized in terms of phase. D: Synthetic LFP spectrograms. The network started out in the ground state and entered an active state after 2 seconds due to stimulation. The signal was produced from 30 pyramidal cells entering foreground (left) and background (right) respectively. The average signal from 5 runs is plotted.

**Figure 3:**
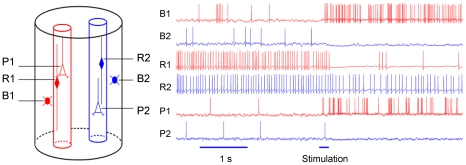
Intracellular potential traces of pyramidal cells and inhibitory interneurons in a simulated hypercolumn. To the left is a sketch of a hypercolumn, where the red minicolumn is in foreground and the blue is in background state. The voltage traces to the right are taken from the same simulation that yielded the raster plot in Figure 2. They show how the neurons behave as the network switches from ground state to a persistent active state (indicated by horizontal stimulation bar). The two upper voltage plots show basket cells, B1 and B2, adjacent to red and blue minicolumn respectively. Middle voltage traces show RSNP neuron membrane potential. R2 is far away from the active minicolumn and maintains an firing rate (although lower than in the ground state). R1, located in the active minicolumn, will fire at a low rate activated only by the low activity of background pyramidal cells. P1 is a pyramidal cell that ends up in the foreground after stimulation, and P2 becomes part of the background.

Similar to other working memory network models in the literature [8], [30], our network could operate in a bistable regime, i. e. two qualitatively distinct activity states co-existed as stable, self-maintained states of the network. Each of the active states engaged a specific subpopulation of the network belonging to the same attractor as elevated-rate “foreground cells” and the remaining neurons as low rate “background cells”. To compare with data from dorsolateral prefrontal cortex [3], [5] obtained in visual delayed response experiments, we mapped our model's ground state to the fixation period activity, and the foreground cells to the cells representing a preferred cue during delay activity. Pyramidal cells in the background were related to those cells that represent a non-preferred cue. Figure 2A, B shows pyramidal cells switching from ground state to a specific active state, with some cells ending up in the foreground, and a majority of them in an even lower rate background activity. Figure 3 shows typical intracellular potential (V_m_) traces of the three cell species as this switch to active state occurs. The firing on the population level is oscillatory in both states, with increased frequencies in the active state moving from beta (20–25 Hz) to gamma band (40–50 Hz). These oscillations are clearly seen in the average V_m_ of a local neuron population and in a synthetic local field potential (LFP). Additionally, the single cell spike-trains were highly variable in all states in accordance with experiments [5]. The spectral power was enhanced during the delay period, and selectively for the foreground population (Figure 2D).

In the following we show that due to the hypercolumnar structure of our network model the bistable range is much larger than in previous models and activity is oscillatory in both states without slow excitatory currents. Further, since inhibition and excitation is approximately balanced in the oscillatory regime, low-rate and high variability of individual spike-trains in all states is achieved without fine-tuning. Several other features such as a very small gap in firing frequencies between the two states, increased oscillation frequency in the active relative to the ground state and correlation between oscillation frequency and firing rate naturally emerges. How these findings relate to each other and the mechanisms behind them will be investigated in subsequent sections.

### Hypercolumns allow bistable oscillatory activity

One problem with existing models of bistable network states is a narrow range of parameter values that give rise to bistability for biologically plausible neuronal firing rates. Our modular network model had a very comfortable bistable range compared to other non-modular models [8]. We define here the bistable range such that its lower boundary is the limit where recurrent excitatory synapses are strong enough to support self-sustained active states, and its upper boundary occurs when this excitation is so strong that the ground state becomes unstable and attractors activate spontaneously. In order to find out if the high stability was due to the network architecture we gradually transformed the network towards a non-modular one. All results in this section were produced without basket-basket cell connections for easier comparison with other models. Since there would be no input to the RSNP cells in the uni-modular case, we temporarily removed RSNP cells also in the multi-modular networks to make a fair comparison.

Most cortex models in the literature feature only one inhibitory population that provides negative feedback to the entire excitatory cell population. In contrast, our model had several hypercolumns defined by the extension of separate populations of basket cells. We gradually decreased the number of hypercolumns, and increased the number of pyramidal cells in each minicolumn to hold the number of neurons in the foreground population constant (Table 1). When comprising only one large hypercolumn the network dynamics resembled that of previous models [8]. A small bistable range existed but the active state would often spontaneously fall back into the ground state during 3 seconds of simulation and the bistable range was hence somewhat ill-defined.

**Table 1:** Bistable range as a function of number of hypercolumns.

Number of Hypercolumns (pyramidal cells/minicolumn)	1 (270)	4 (67)	9 (30)
**Bistable range**	1.30–1.43	0.89–1.29	0.75–1.20
**Bistability ratio**	1.10*	1.45	1.60
**Active state stability**	10/20	20/20	20/20
**Frequency range**	21–23 Hz	22–25 Hz	22–29 Hz
**Mean firing rate** **(s^−1^)**	0.46–0.54	1.6–2.6	2.8–5.5

*Bistable range* is the level of maximum and minimum recurrent excitatory conductance allowing for bistability, where 1 is the standard value; *bistability ratio* is upper boundary/lower boundary; *activity state stability* is the fraction of active states that are stable for 3 seconds or more in the middle of the bistable range; *frequency range* refers to the oscillation frequency of V_m_ in the active state in the bistable regime (faster oscillations for stronger excitation). The oscillation frequency of the ground state was ∼20 Hz in all simulations; *mean firing frequency* is the average firing frequency of foreground cells at the lower and upper boundary of the bistable regime.

The low gap in oscillation frequencies between ground and active state in the uni-modular case meant that a small perturbation in activity allowed background cells to start fire and the active state to transition back to the ground state. If feedback inhibition was lowered stable solutions existed, but they were non-oscillatory and the bistability ratio was very small (1.02).

**Figure 4:**
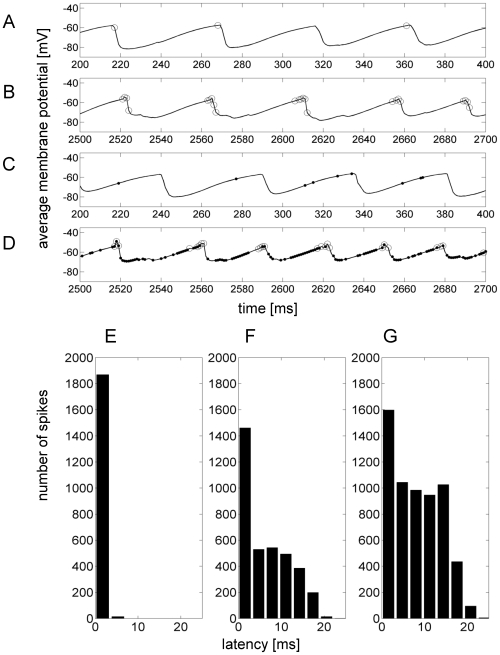
Out-of-phase excitation. Upper panel (A–D): Shows average voltage of one minicolumn and the spiking output within (circles) and from other connected (dots) minicolumns. A is taken from the ground state in a one-hypercolumn network, B from the active state in the same network. C from the ground state in a nine- hypercolumn network, D from the active state. Lower panel (E–G): Spike histogram showing spike latency to the nearest membrane potential peak. In the one-hypercolumn case (E) all excitatory input arrives around the peak while in the case with four (F) and especially nine (G) hypercolumns incoming excitation is more distributed in time.

As the number of hypercolumns was increased, active states became stable at lower levels of recurrent excitation and spontaneous termination of an active state never occurred. Long-range pyramidal to pyramidal cell excitation between hypercolumnar modules arriving out-of-phase with the local oscillations (Figure 4) stabilized the persistent activity in the active states. The firing and oscillation frequencies in the active states increased (keeping recurrent excitation constant) with the number of hypercolumns. This could be explained by the fact that a similar number of pyramidal cells in a minicolumn fired in each oscillatory cycle, regardless of the minicolumnar size (Figure 4 B, D). Thus, more cells were allowed to spike with increased modularization thereby increasing the total amount of recurrent excitation and producing shallower but equally steep V_m_ deflections, leading to an increased oscillation frequency. Since destabilizing the ground state in modular networks required the simultaneous activation of a larger number of cells, less likely to happen by chance, the upper boundary of the bistable range did not decrease as much as the lower one. Thus, the width of the bistable range increased with modularization.

The most pronounced effect of having the RSNP cells in this network was a shift in the bistable range towards larger recurrent excitatory conductances (along with a slight increase of the range). As oscillation frequency increased with recurrent excitation this therefore increased the oscillation frequency in the active state to between 29 and 42 Hz in the 9 hypercolumns case, while the oscillation frequency in the ground state increased from ∼20 to ∼21 Hz. With RSNP cells the network better matched the gamma-frequency increase seen during delay [4] in working memory tasks.

As mentioned previously, it is possible to stabilize persistent activity in a recurrent network in the oscillatory regime with e.g. NMDA-synapses [20]. It has even been argued that this is the only way to achieve robust oscillatory persistent activity [19], [31]. When NMDA synaptic currents were blocked in our network, persistent activity was preserved if this was compensated for with enhanced AMPA conductances. Additionally, if the external noise input was removed the network could still engage in persistent activity if the amount of AMPA-mediated recurrent excitation was further increased. Conserving either long-range inhibition or long-range excitation was sufficient. Thus, having several connected hypercolumns oscillating out of phase replaced the effect of a slow excitatory current and did not require fine-tuning. This allowed us to attain a robust bistable oscillatory regime which resulted in several interesting and novel features of the model as further explained below.

### Oscillatory regime is balanced and show irregular and low rate firing

Existing attractor memory models face three major issues; 1) the persistent activity state has much higher firing rates than the ground state at odds with most experimental data, 2) high firing rates result in lost balance between excitation and inhibition, and 3) as a consequence the firing in the active state becomes regular. Problems 2) and 3) have been extensively studied [14]–[16] and a solution to 3) was recently presented [16]. We find that the modular network investigated here provides a solution to all three problems.

Regarding 1), in our model the average firing rate in the ground state was 0.5–1 s^−1^. In the active state, background cells fired at an average of ∼0.1 s^−1^ and this state was stable if the foreground population fired from about 3 s^−1^ (without RSNP inhibition) at the lower boundary of the bistable range. Thus, the firing rate in the ground state was consistently lower than in the foreground population of the active state but the gap between the two was small; with a minimum gap around 2–3 s^−1^ close to the experimental values reported [5]. However, with the standard network architecture (with RSNP inhibition) and set of parameters given in the Methods and Supplementary material (Text S1), average firing rate was 15 s^−1^ due to stronger recurrent excitation (middle of bistable range).

**Figure 5:**
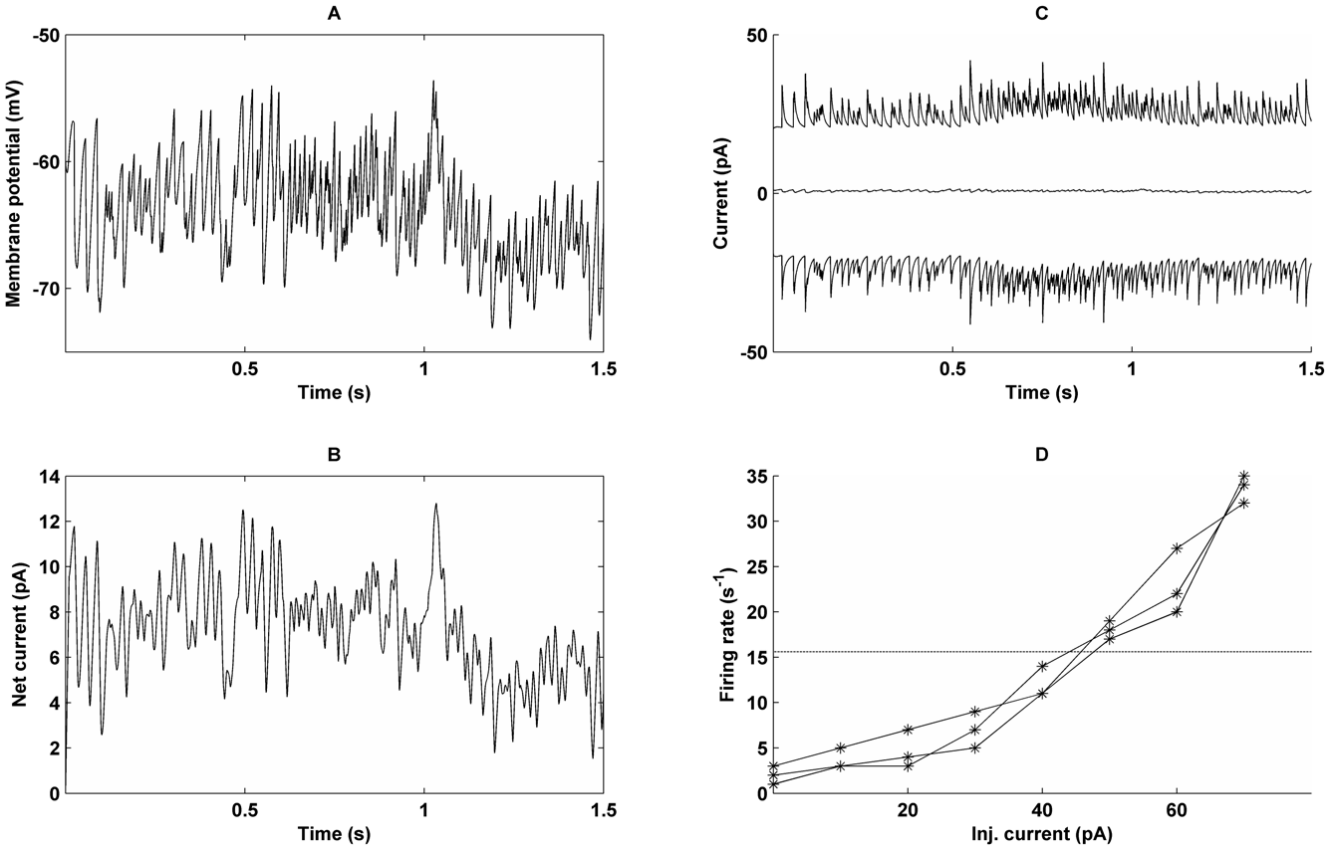
Balance of currents in a single cell during three different models of activity of the network. From 0–0.5 s network is in its ground state, from 0.5–1.5 s it is in an active state. Between 0.5–1 s the cell is part of the foreground and between 1–1.5 s it is part of the background. A: Plot of the soma potential. Note that the soma was injected with a negative current (−0.2 nA), so that the cell did not spike while we measured the balance of currents. B: Net, i.e. total excitatory+inhibitory, currents into soma. C: Top line is the excitatory current into soma, and the bottom is the inhibitory current that almost perfectly balances the excitatory one. The middle line is the net synaptic current of panel B, the result of imbalance between excitatory and inhibitory currents. Notice its significantly smaller amplitude. D: Plot of firing frequency as a function of current injected into the soma in three different cells in ground state. The dashed line corresponds to the mean firing rate in the active state in the same network.

Regarding 2) we investigated the balance of currents in the oscillatory regime, since such a balance between excitation and inhibition produces highly irregular firing [12], [13]. We measured the currents into the soma (as described in Methods) on a cell injected with a hyperpolarizing current to prevent it from spiking, as it participated in the different states of the task (Figure 5A–C). The net current into the soma was slightly excitatory in both the ground state and in the foreground of the active state, ∼7.7 pA and ∼8.0 pA respectively. When we disabled the recurrent connectivity we found that the net excitatory contribution from noise synapses (representing input from cells outside the simulated network, see Methods) was approximately 10 pA, implying that the net contribution from the recurrent network was inhibitory for both ground and active state. The mean firing frequency with recurrent connections removed and only noise driving the cells was ∼8 s^−1^ compared to ∼15 s^−1^ with the recurrent network enabled. This shows that increased spiking rates in the foreground cells were driven by other mechanisms than a net increase in excitation. In addition, we injected current into a cell during the ground state (Figure 5D). A cell firing at 1–3 s^−1^ without current injection required between 30 and 40 pA to reach 10 s^−1^. This is about 100 times the difference in mean net soma current between cells in the foreground and those in the ground state in the fully functional network, again demonstrating that net increase in excitatory current was not a direct cause of the increase in firing rates.

**Figure 6:**
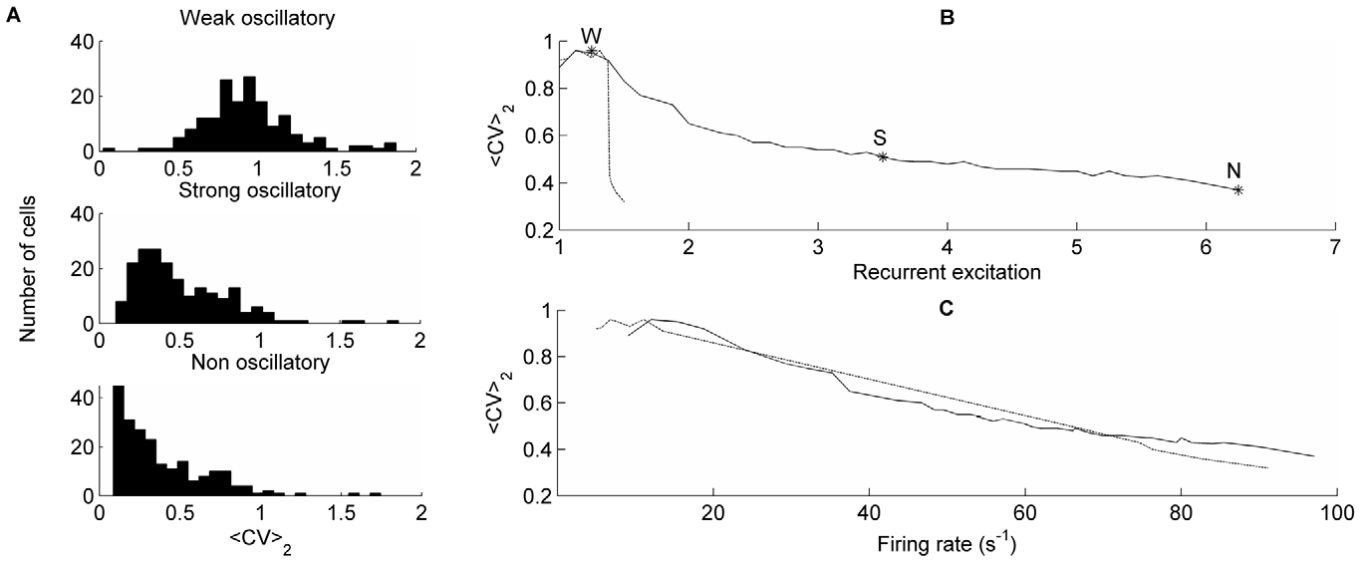
<CV_2_> and oscillations. A: <CV_2_> histograms for the foreground pyramidal cells in the active state for three different networks, displaying weak, strong and non-oscillatory activity. We move between the different networks by manipulating the level of recurrent excitatory conductance. B: Relative recurrent excitatory conductance vs <CV_2_>, where weak (“W”), strong (“S”) and non-oscillatory (“N”) networks are marked. Recurrent excitatory conductance = 1 is defined as the smallest possible recurrent excitatory conductance for which we had stable memory retrieval. The solid line is for a network with basket to basket cell connections, the dashed line corresponds to the same network, but with no basket to basket cell connections. Note that we here go outside the bistable range (Table 1) for high levels of excitation. C: <CV_2_> against average firing rate. The solid line represents the network with basket to basket cell connections, the dashed line the one without such connections.

To investigate 3) above, we measured the local ISI-variability (Figure 6A), <CV_2_>, as described in the Methods section. <CV_2_> was high (0.8–1.1) in all states throughout the bistable range and therefore required no fine-tuning. For the standard parameters, with basket to basket cell connectivity enabled, <CV_2_> was 0.96 in foreground and background pyramidal cells and 0.98 in ground state. Without basket to basket cell connections the variability was slightly lower, especially in the ground state. To rule out that Poisson noise was the main source of high variability we measured the variability of pyramidal cells only driven by such noise, and found a <CV_2_> of 0.69. The modeled basket cells showed a high variability (<CV_2_> of 0.89) in both ground and active states, whereas RSNP cells typically had lower variability, with <CV_2_> increasing from 0.25 to 0.45 respectively.

In Figure 6B we plot the <CV_2_> against the level of recurrent excitatory conductance for the network with and without basket to basket cell connectivity. When these connections were disabled <CV_2_> was stable and quite high in the oscillatory regime and drastically dropped as the excitation was increased beyond the bistable range to the level where firing became asynchronous (leaving the inhibition dominated regime). This dramatic shift and drop in variability was not seen with basket to basket cell connectivity enabled, but instead there was a more continuous transition to the asynchronous regime. The different sensitivity of neuronal dynamics to recurrent excitation in these two cases could be explained by the effect it had on neuronal firing rate (Figure 6C).

Since oscillations of different frequencies are prominent both in resting and active cortical states *in vivo* [4], [32]–[35] and *in vitro* [36], we next studied oscillation frequency modulations in the different states.

### Oscillation and firing frequency modulations

As mentioned, the frequency and spectral power of beta-gamma band oscillations increased when switching from ground to active state. This is supported by strong correlation with pronounced power enhancement in gamma-band with delay activity [4], [33] and with increased firing rates [35]. This frequency increase was robust when changing *τ_GABA_* from 6 to 25 ms and changing the reversal potential from −85 mV to −70 mV, though oscillations were generally slower with longer GABA time constants. With disabled basket to basket cell connections the oscillation frequency decreased from ∼20–25 Hz to ∼15–20 Hz in ground state and from ∼40–50 to ∼30–40 Hz in the active state. This general shift in both states towards lower frequencies is in agreement with previous results of a more abstract model [37] and it also explains the shift in frequencies compared to our previous models [17], [38] where basket to basket cell connections were not present.

**Figure 7:**
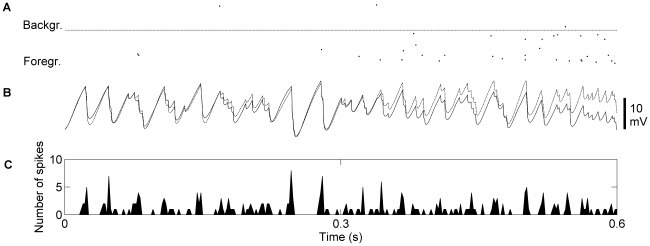
Spiking activity and soma potentials of pyramidal cells in foreground and background. A: Pyramidal cell spike output from two minicolumns, one entering the foreground of the active state (bottom), the other entering the background of the active state (top) at t = 0.3 s. B: Mean soma potentials of the same two minicolumns. The mean potential of the foreground cells (dashed) is systematically above the mean potential of the background cells (solid). Firing threshold is marked with solid horizontal line. C: Summed pyramidal cell spike output of one hypercolumn. The number of spikes within the hypercolumn in ground state (0–0.3 s) and active state (0.3–0.6 s) stayed almost constant. Measured over longer intervals the total spike output was slightly lower in active state.

Next we studied the mechanisms behind the increased firing rates in the active state compared to the ground state. It was not due to a net increase in excitation (see above) or to an increase in fluctuations [15] since soma potential variability decreased slightly in the active state. We found that in each oscillatory cycle there exists a time window, where pyramidal cell membrane potentials climb close to the firing threshold (Figure 7). If and when in this window of opportunity a cell fires is decided by fluctuations and small biases in net input between competing populations. Firing rate increased in the active state in two different ways; firstly the frequency of population oscillations increased around 40% when entering the active state. Secondly, the foreground cells spiked with an increased probability in each oscillatory cycle, from ∼1 spike in 25 oscillations to ∼1 in 3 oscillations (Figure 7 A–B). The increased probability of firing per oscillatory cycle in the foreground cells occurred since they had a systematically slightly higher membrane potential relative to background cells and therefore consistently reached threshold first. With similar feedback inhibition in ground and active state this requires that active state oscillations are faster than ground state oscillations since background cells have essentially the same excitation in the two cases. This “racing condition” between foreground and background cells during oscillatory activity has previously been referred to as a “winner-takes-all algorithm” [33]. It allows for fast transitions between attractor states since small differences in excitation are sufficient to switch foreground subpopulation [13]. Figure 7C shows that feedback inhibition in the network regulates total pyramidal cell activity such that the number of spikes remains almost the same in ground and active states. However, in the active state the firing is confined to a small fraction of the cells which fire at an elevated rate (the foreground population), whereas in the ground state, spiking is distributed among all the pyramidal cells in the network.

## Discussion

We have proposed and investigated a cortical network model of working memory featuring a wide bistable range, oscillatory population activity, and low-rate irregular neuronal spiking patterns. The single most distinguishing structural property of the model was its modularization in terms of hypercolumns interacting via long-range excitatory synapses terminating on pyramidal neurons or dendritic targeting inhibitory interneurons. The persistent active state was stable for rates as low as ∼3 s^−1^ in the foreground cells which is remarkably low and yields a small gap between ground and active states much closer to experimental data [5] than previous spiking working memory models. In terms of dynamics, population activity was oscillatory in ground as well as in active states with increased power in gamma-band during an active state [4], [33] and for elevated firing rates [35]. Our results indicate that such increase in oscillation frequency is required in order to have bistability in the oscillatory regime, and is helped by the modular structure and specific inhibition from RSNP cells which both significantly increased the gap in oscillation frequency between the two states.

In addition to the modular structure the presence of NMDAR gated synapses [20] also stabilized oscillatory activity. But in contrast to previous results [18], [19], [31], our network showed persistent oscillatory activity even with NMDAR blocked. Bistability remained over a large range of recurrent excitatory conductance and was also observed in previous studies with more pronounced cellular adaptation and synaptic depression [17], [38]. This result points toward computational advantages with a modular structure and the need for large-scale network models that span more than just the local network.

While cellular adaptation was present in the model it was reduced relative previous work with quasi-stable attractors [17], [38]. This allowed comparison with persistent state models which assume delay activity to be a stationary state. Whether delay activity *in vivo* is indeed persistent or is switched on and off in slow theta-like oscillations as is the case with stronger adaptation is still unclear [7], [39]. In the latter case the gaps in firing rates between ground state, background and foreground activity would decrease even further as foreground rates would decrease and background rates increase. The same would be true if interneurons were included in the data [5], as they had similar or even decreasing rates in the active state in the model. Further, calculating firing rates as the mean of inverse ISI:s over some time interval as is often done experimentally, would also give a bias towards higher background rates.

*In vivo* data shows high ISI-variability both during fixation and delay period activity in working memory tasks [5], [7]. Data seems to indicate that variability during the delay period can both increase [5] and decrease [7] depending on cortical area studied. Previous models have demonstrated that oscillatory activity can produce highly irregular spike output [21], [37], [40] and our model operated in this regime in the ground as well as active state. Bistable, irregular firing has previously been studied in the non-oscillatory regime [14]–[16]. While two models [14], [15] required fine-tuning, a recurrent network model with near-threshold post-spike voltage reset and depressing synapses did not [16]. In our model the bistable range was however much larger (60% compared to ∼9%) and it was stable with a much smaller gap in firing rates between the two states.

Another interesting result of the winner-takes-all dynamics in the oscillatory regime is that two minicolumns of different size, everything else being equal, produces the same output in terms of numbers of spikes. This implies that such a system is very robust to cell loss or variations in functional column size which might be important for large-scale coordination.

Our model is admittedly complex when it comes to component neuron and synapse models as well as architecture and connectivity. Some aspects of its architecture and connectivity remain hypothetical like, for instance, the specific long-range innervation of RSNP cells. The behavior of the model depends critically on the network architecture and connectivity but, as already mentioned, preliminary results indicate that a similar behavior could be reproduced in networks with less complex model neurons and synapses.

In conclusion, our results indicate that the bistable oscillatory regime has interesting properties and that modularization of a cortical network model is important for the type of dynamics it displays. We demonstrate that depending on whether or not there is a prominent synchrony at the time-scale of the gamma period significantly affects the fit to experimental data on cortical firing patterns and population oscillations. Our results suggest that a lack of synchrony at millisecond timescales between gamma oscillators is, in fact, important for stabilizing global cortical activity states. This is at odds with current theories of phase locking of gamma oscillations over longer distances [32] and further experimental and large-scale modeling studies are required to achieve a more coherent understanding of these phenomena.

## Methods

### The network model

We used a biophysically detailed network model of cortical layer 2/3, which was developed previously [17], [38], [41]. It had both a hypercolumnar and a minicolumnar organization (Figure 1). Neurons within a hypercolumn were organized in 49 non-overlapping subpopulations (minicolumns). The network was composed of 9 such hypercolumns. Such a columnar organization has anatomical [42] and functional [43] support in data from prefrontal cortex. The minicolumns were spread out on a two-dimensional square grid with a 1.5 mm side and each minicolumn had a diameter of 30 µm. All pyramidal cells of a certain minicolumn shared the same x and y coordinates but where uniquely spread out on the z-axis along 500 µm. Interneurons were placed near the center of each minicolumn with respect to the z-axis.

The cells included were layer 2/3 pyramidal cells and two different types of inhibitory interneurons, assumed to correspond to fast spiking, horizontally projecting and soma targeting basket cells and regular spiking, vertically projecting and dendrite targeting cells (RSNP), e.g. double bouquet cells [25], [27]–[29]. Each minicolumn contained 30 pyramidal cells [22], [23], one basket cell and two RSNP cells [24]–[26].

### Connectivity

As in previous studies, the connectivity was set up to store non-overlapping memory patterns, here 49 different patterns, each comprising 9 equal-selectivity minicolumns in different hypercolumns. However, recent studies have demonstrated that also overlapping patterns can be stored robustly in this network (unpubl. obs.).

Pyramidal cells in a minicolumn connected to 25% of the other pyramidal cells in their own minicolumn [44] as well as to the eight closest basket cells in their own hypercolumn. The rest of their connections targeted pyramidal cells or RSNP neurons in other hypercolumns. The RSNP cells projected locally to the dendrites of the pyramidal cells in their own minicolumn. They provided disynaptic long-range inhibition from minicolumns of dissimilar selectivity in other hypercolumns. Basket cells provided feedback inhibition targeting the cell soma of 70% of all pyramidal neurons within their hypercolumn non-selectively. Each basket cell also connected to 70% of the other basket cells in the same hypercolumn. *In vivo* chemical synapses between basket cells seem abundant [45]. We did not have gap junctions between the inhibitory interneurons in our model. We studied the network both with and without such basket to basket cell connectivity. Connections between pairs of neurons were randomly generated according to the connection densities. A connection was always carried out by a single bouton and all connections of a neuron onto itself were removed.

For all local connections within a hypercolumn, we constrained the network connectivity and EPSP sizes with biological data, mostly from Thomson et al. [44]. For long-range connections there is a relative lack of data, as this type of connectivity is quite difficult to measure quantitatively. We therefore extrapolated the available experimental data, based on theoretical considerations, to arrive at a plausible amount of global excitation. From levels of activity and total number of pyramidal synapses onto a layer 2/3 pyramidal cell we estimated the number of connections from other active pyramidal cells to be in the order of one hundred. This resulted from the fact that a layer 2/3 pyramidal cell receives on average some 10 000 synapses and that roughly 1% of pyramidal cells in cortex fire at an elevated rate while the rest are almost silent [46]–[48], which gives at least (for totally non-specific connectivity) around one hundred active synapses from other pyramidal cells onto a layer 2/3 pyramidal cell. Out of these, less than ten (assumed 25% connectivity [44] and 30 cells in the layer 2/3 portion of a minicolumn [22], [23]) on average are from within the cell's own minicolumn. The connections originating outside the local minicolumn were thus estimated to be about ten times more numerous, but interestingly their EPSPs are also significantly weaker than local ones [49]. In the current model the local to global EPSP were 3∶1 in magnitude. A pyramidal cell had 90 excitatory synapses from other distant pyramidal cells with equal selectivity as the minicolumn it was part of. An RSNP cell had 30 excitatory synapses from distant pyramidal cells for each pattern with different selectivity than its own minicolumn. Using these figures implied that for the foreground pyramidal cells in an active state, global and local excitation was approximately balanced.

### Neuron model

Our model neurons were multi-compartmental and conductance-based, following the Hodgkin-Huxley and Rall formalisms. Pyramidal cells consisted of 6 compartments (soma, basal dendritic, initial segment, and three apical dendritic) and interneurons of 3 (soma, dendritic, and initial segment). The potential in a compartment was calculated by integrating the currents
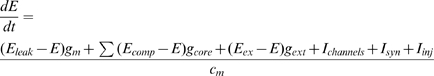
(1)where *c_m_* is the capacitance of the membrane, *g_m_* is the membrane leak conductance, *E_leak_* is the equilibrium potential of the leak current, *g_core_* is the core conductance between connected compartments, which is dependent on compartmental cross section (equal for basal and apical dendrites, smaller for initial segment). *g_ext_* is a non-specific excitatory conductance with reversal potential *E_ex_*, *I_channels_* is the active currents from the different ionic channels in the membrane of the compartment, including voltage-dependent Na^+^, K^+^, and Ca^2+^ channels as well as Ca^2+^-dependent K^+^ channels. *I_syn_* is the current through glutamatergic and GABA-ergic synapses on the compartment and *I_inj_* is injected current.

Parameters were tuned to mimic the spiking behavior of the respective neuron type. Pyramidal cells were strongly adapting, basket cells almost non-adapting and RSNP cells had intermediate adaptation. For individual cells of a certain type all parameters were fixed except size, which varied ±10% according to a uniform distribution. This introduced a moderate variability in cell excitability. A detailed account of all ionic channel equations and parameters used is further given in the supplementary information (Text S1).

### Synapse model

The pyramidal to pyramidal and pyramidal to RSNP connections had both AMPAR and voltage dependent NMDAR components. Synapses formed by pyramidal cells onto basket cells were purely AMPAR-mediated while the inhibitory cells formed GABA_A_ type synapses. Excitatory inputs (including noise) were placed on the second apical and on the basal dendritic compartment, while the inhibitory basket cells connected to the soma. The inhibitory synapses from RSNP cells connected to the second apical dendritic compartment. The synapses formed by pyramidal cells were fully saturating in the sense that the conductance *G_syn_* during repetitive firing could only sum up to the peak conductance resulting from a single presynaptic spike. In order to allow comparison with a delayed match to sample task, where memory attractors were expected to be stable for several seconds, synaptic depression between pyramidal cells included in the original model was disabled in the simulations performed here. After a synaptic event, conductance decays back to zero with a time constant τ*_syn_* that is characteristic of each type of synapse. Here, τ*_AMPA_* = 6 ms, τ*_GABA_* = 6 ms, as AMPA and GABA_A_ has been reported to have similar time constants [50], and τ*_NMDA_* = 150 ms. Reversal potential was zero for AMPA and −85 mV for GABA. Robustness was tested for τ*_GABA_* up to 25 ms and a GABA reversal potential up to −70 mV. The axonal conduction speed was 0.2 m/s and the synaptic delay 0.5 ms. Since model neurons operated at low rates (less than 3% of inter-spike intervals between 6–12 ms), the AMPA saturation did not play an important role and could be removed without affecting the results.

### Noise and input from layer 4

Pyramidal cells received noise input through excitatory AMPA synapses activated by simulated Poisson spike trains with an average firing of 300 s^−1^ but with very small conductances (0.08 nS, ∼10 times smaller than local pyr-pyr conduction). This source alone made the pyramidal cells spike at ∼8 s^−1^. Single minicolumns could be selectively stimulated by pyramidal cells mimicking layer 4 input cells. Each minicolumn had five such cells and these were activated (60–100 s^−1^) by spike trains generated by Poisson processes and innervated the 30 layer 2/3 cells with feedforward connections (50% connectivity). This setup was found adequate for activating our layer 2/3 model, though more elaborate models of layer 4 to 2/3 connectivity exist [51].

### Activity

By modulating the level of overall inhibition we made the network bistable [8]. Therefore, it did not engage in specific activity spontaneously, as reported previously [17], but stayed in the ground state until a memory fragment was stimulated and the corresponding complete memory pattern activated. The retrieved memory was then maintained active through recurrent excitation until adaptation mechanisms (if strong enough) terminated the attractor. . In order to allow comparison with a delayed match to sample task we promoted persistent activity in memory attractors by a decreased NMDA calcium influx (15% of what was used in the original model [21]) to reduce calcium-dependent adaptation currents.

### Testing bistability

We used the layer 4 input to test the range of bistability of the model in the following fashion: First we simulated 3 seconds of activity to test that the ground state was stable, then we added stimulus for 300 ms on a fraction of the minicolumns in a pattern (5 out of 9) to see if the pattern was completed. After that followed a 3 second period with no stimulus to see if the pattern was stable (3 seconds is much longer than any adaptive mechanism in the network). If the network passed this test with up to twenty different random seeds it was said to be bistable for the specific parameters.

### Measuring balance of currents

Since we had a multi-compartmental cell model the balance of excitation and inhibition could not be measured simply as the currents through synapses (which were spread out on the different compartments). We had to take into account all current influx to the soma. During persistent activity when the dendritic targeting RSNP cells in the foreground minicolumns were almost silent, the influx from dendrites was dominantly excitatory, but in the ground state this influx was a mixture of excitation and inhibition. Using a method modified from [52] the inhibitory current was calculated by setting the sum of all current fluxes into the soma to zero,
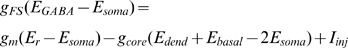
where *I_inj_* is a negative current injection into soma to keep the cell silent and *g_FS_*, the conductance through the basket cell synapses, is the only unknown. The first term on the right hand side is the leak current and the second term represents the current influx from the proximal dendritic compartment.

### Simulation

We used the SPLIT simulator [53], a simulator developed for simulations of large, biophysically detailed network models which can run on a single processor as well as on massively parallel machines. The model presented has been scaled up to the size of 22 million neurons and 11 billion synapses on a Blue Gene/L supercomputer [38]. The network simulated here consisted of 14553 cells and 1.8 million synapses. Simulations were typically performed on 128 nodes of the Blue Gene/L computer at the Center for Parallel Computers at KTH. It took 81 seconds to simulate one second of network activity.

### Synthetic LFP

LFP was estimated by calculating the potential difference between the soma and the first dendritic compartment of all cells in a local population in each time step, similar to a previous method [54]. This method relies on the assumption that dipoles are the main source of the LFP signal, and that they are produced by currents in the geometrically aligned apical dendrites of pyramidal cells. The average of this signal was then subtracted before a spectrogram was produced in Matlab.

### Statistical analysis

From the series formed by the inter-spike intervals (ISIs) of each spike train we computed a local measure of ISI variability, CV_2_. CV_2_ is computed by comparing each ISI (ISI_n_) to the following ISI (ISI_n+1_) to evaluate the degree of variability of ISIs in a local manner [55]:
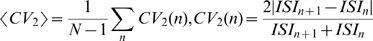
A Poisson spike train has a CV_2_ of 1. The CV_2_ measure was used since it is not rate dependent in the dynamical range of the network, as the CV measure turned out to be (giving lower variability for low rate Poisson spike trains).

## Supporting Information

Text S1 Model equations.
(0.16 MB DOC)
