# Supplementary material for: Bistable, Irregular Firing and Population Oscillations in a Modular Attractor Memory Network
Source: PLoS Comput Biol. 2010 Jun 3;6(6):e1000803. doi: 10.1371/journal.pcbi.1000803 (PMC2880555; doi:10.1371/journal.pcbi.1000803)
Supplement: Text S1 — Model equations. (0.16 MB DOC) [file pcbi.1000803.s001.doc]

**Appendix A – model equations**

*A1. Membrane potential*

The neurons are based on a previously developed formalism [1] and cell parameters set to mimic FS and RS cells [2]. The modelled neurons are represented by 3-6 electrically coupled isopotential compartments. The potential *E* is computed according to the Hodgkin-Huxley formalism by calculating the ionic current flux into the compartment. The potential in a compartment is calculated by integrating the currents

$\frac{dE}{dt}=\frac{(E_{leak}-E)g_{m}+\sum(E_{comp}-E)g_{core}+(E_{ext}-E)g_{ext}+I_{inj}+I_{channels}}{g_{m}}$ (1)

Here *c_m_* is the membrane capacitance proportional to the membrane area and $(E_{leak}-E)g_{m}$ is a passive leakage current with conductance *g_m_* across the membrane and *E_leak_*  is the equilibrium potential. The next term in the nominator is the contributing currents from electrically coupled compartments with potential *E_comp_* and the conductance *g_core_*. Further, $(E_{ext}-E)g_{ext}$ is the current from diffuse transmitter stimulation setting the membrane resting potential and *I_inj_* is injected current. The last term is the active currents from the different ionic channels in the compartments membrane (see A2). This actively mediated current is described by several Hodgkin-Huxley-type equations given below. The Hodgkin and Huxley model describes how the mean conductance through the sodium and potassium channels changes with the membrane potential. The sodium channel current is given by

$I_{Na}=(E_{Na}-E)g_{Na}m^{3}h$ (2)

Where *g_Na_* is the maximum sodium conductance and *m* and *h* is the voltage-dependent degree of activation and inactivation of the sodium channels. Each channel is either on or off but their activation as a population is modelled. The activation, *m*, of sodium channels is given by

$\frac{dm}{dt}=\alpha_{m}(1-m)-\beta_{m}m$ (3)

Where α is the rate at which channels switch from closed to open state and β is the reverse. They depend on the membrane potential

$\alpha_{m}=\frac{A(E-B)}{1-e^{(B-E)/C}}$ $\beta_{m}=\frac{A(B-E)}{1-e^{(E-B)/C}}$ (4)

And A, B and C are constants. The inactivation of sodium channels is given in a similar fashion

$\frac{dh}{dt}=\alpha_{h}(1-h)-\beta_{h}h$ (5)

$\alpha_{h}=\frac{A(B-E)}{1-e^{(E-B)/C}}$ $\beta_{h}=\frac{A}{1-e^{(E-B)/C}}$ (6)

The potassium channels have no inactivation

$I_{K}=(E_{K}-E)g_{K}n^{4}$ (7)

$\frac{dn}{dt}=\alpha_{n}(1-n)-\beta_{n}n$ (8)

$\alpha_{n}=\frac{A(E-B)}{1-e^{(B-E)/C}}$ $\beta_{n}=\frac{A(B-E)}{1-e^{(E-B)/C}}$ (9)

An exponential of four is used in eq (7) in order to achieve delay of activation of potassium channels as not to cut of the spikes prematurely. The model described by these equations is able to produce action potentials with realistic shape.

With the addition of calcium dependent potassium channels

$I_{K(Ca)}=(E_{K}-E)g_{K(Ca)}([Ca_{AP}]+[Ca_{NMDA}])$ (10)

which provide adaptation, realistic rates of repetitive firing is also achieved. As is evident from this equation there are two sources of calcium influx. One source is voltage dependent calcium channels also following a Hodgekin-Huxley dynamics

$I_{Ca}=(E_{Ca}-E)g_{Ca}q^{5}$ (11)

$\frac{dq}{dt}=\alpha_{q}(1-q)-\beta_{q}q$ (12)

$\alpha_{q}=\frac{A(E-B)}{1-e^{(B-E)/C}}$ $\beta_{q}=\frac{A(B-E)}{1-e^{(E-B)/C}}$ (13)

which activate at high potentials, mainly after spikes. The calcium entering through these channels is denoted Ca_AP_ and is filling up a separate pool as compared to the calcium entering through NMDA type synaptic channels (see section A2). The reason for this is that the time constants for the build-up and decay of calcium levels in the two different processes differ. The Ca_AP_ influx is proportional to the calcium current. The modelled calcium concentration decays exponentially which gives

$\frac{d[Ca_{AP}]}{dt}=(E_{Ca}-E)\sigma_{AP}q^{5}-\delta_{AP}[Ca_{AP}]$ (14)

Where σ_AP_  is the rate of calcium influx and δ_AP_ is the rate of decay. The NMDA-channel is not only open to sodium and potassium but also calcium. This calcium builds up slower than that from voltage gated calcium channels and is responsible for adaptation on longer time scales in the model. The Ca_NMDA_ concentration is given by

$\frac{d[Ca_{NMDA}]}{dt}=(E_{nmda}-E)\sigma_{NMDA}ps-\delta_{NMDA}[Ca_{NMDA}]$ (15)

Where s is the activation of the channel and p, ranging between 0 and 1, models the NMDA magnesium block. The calcium ion influx rate is given by $\sigma_{NMDA}$.

*A2. The synapses*

Glutamatergic synapses of AMPA or NMDA type and GABAergic synapses share the same equations but differ in time-constants and reversal potentials (Table A2). After the initiation of a presynaptic spike, following a delay set proportional to the neurons distance plus a constant for the transsynaptic delay, the conductance is increased step-wise to its maximum value (s in eq. 16 is set to 1). It then decays back to zero exponentially with some decay time constant.

$I_{syn}=(E_{syn}-E)g_{syn}s$ (16)

Here s, the activation level, gives this box-shaped function. E is the potential of the postsynaptic compartment and E_syn_ is the reversal potential of the synapse. The time dependence of s is described by the following equation

$\frac{ds}{dt}=\left\{\begin{matrix} (1-s)/\text{ }\tau_{\text{raise}} & \begin{matrix} if & t-t_{1}<t_{dur} \end{matrix} \\ s/\tau_{\text{decay}} & otherwise \end{matrix}\right.$ (17)

If $\tau_{\text{raise}}$is short compared to the duration of the raising phase the synapse is saturating, reaching full conductance from one presynaptic spike. In our model all synapses were fully saturating. For NMDA-synapses we additionally have a voltage-dependence of the synaptic current

$I_{NMDA}=(E_{NMDA}-E)g_{NMDA}ps$ (18)

where p and s is the same as in eq (15). The variable p is voltage dependent and modelled in a similar fashion as other voltage dependent channels

$\frac{dp}{dt}=\alpha_{p}(1-p)-\beta_{p}p$ (19)

$\alpha_{p}=Ae^{E/C}$ $\beta_{p}=Ae^{-E/C}$ (20)

## **A3 – Cell and synapse parameters**

| **Parameter** | **Pyramid** | **RSNP** | **Basket** | **Unit** |
| --- | --- | --- | --- | --- |
| g_ext_ (from diffuse stimulation) | 0.082 | 0.15 | 0.15 | μS /mm^2^ |
| g_core_  E_ext_ | 8.1  0 | 8.1  0 | 8.1  0 | μS /mm^2^  mV |
| E_leak_ | -75 | -75 | -75 | mV |
| E_Na_ sodium current eq. pot. | 50 | 50 | 50 | mV |
| E_Ca_  calcium current eq. pot. | 150 | 150 | 150 | mV |
| E_k_ potassium current eq. pot. | -80 | -80 | -80 | mV |
| E_Ca(NMDA)_  eq. pot. (NMDA) | 20 | 20 | 20 | mV |
| g_m_ membrane leak conductance | 0.74 | 0.44 | 0.44 | μS /mm^2^ |
| c_m_  membrane capacitance | 0.01 | 0.01 | 0.01 | μF /mm^2^ |
| Soma diameter ± stdev | 21± 2.1 | 7± 0.7 | 7± 0.7 | μm |
| g_Na_ initial segment | 2500 | 2500 | 2500 | μS /mm^2^ |
| g_K_ initial segment | 83 | 5010 | 5010 | μS /mm^2^ |
| g_Na_ soma | 150 | 150 | 150 | μS /mm^2^ |
| g_K_ soma | 250 | 1000 | 1000 | μS /mm^2^ |
| g_noise_ | 0.08 | - | - | nS |
| Noise rate | 300 |  |  | s^-1^ |
| Ca_V_ influx rate | 1.00 | 1.00 | 1.00 | mV^-1^ ms^-1^mm^-2^ |
| Ca_NMDA_ influx rate | 4.44⋅10^8^ | 10.6⋅10^6^ | - | s^-1^mV^-1^μS ^-1^ |
| Ca_NMDA_ decay rate | 1 | 1 | - | s^-1^ |
| Ca_V_ decay time rate | 6.3 | 4 | - | s^-1^ |
| g_K_ (Ca_V_) | 29.4 | 105 | 0.368 | nS |
| g_k_ (Ca_NMDA_) | 6 | 6 | - | nS |
| N:o compartments | 6 | 3 | 3 |  |
| Dendritic compartment area (fraction of soma) | 4 | 4 | 4 |  |
| Initial section area (fraction of soma) | 0.1 | 0.1 | 0.1 |  |

Table A1. Table of neuron parameters.

| Pre-post | Type | t_dur_  s | τ_raise_  s | τ_decay_  s | E_rev_  mV | E_slow_  mV | Syn cond.  nS |
| --- | --- | --- | --- | --- | --- | --- | --- |
| Pyr-Pyr (l/g) | Kainate/AMPA | 0.0 | 0.0 | 0.006 | 0 | - | 0.77/0.34 |
| Pyr-Pyr (l/g) | NMDA | 0.02 | 0.005 | 0.150 | 0 | 0.020 | 1.35/0.6 |
| Pyr-Basket | Kainate/AMPA | 0.0 | 0.0 | 0.006 | 0 | - | 0.09 |
| Pyr-RSNP | Kainate/AMPA | 0.0 | 0.0 | 0.006 | 0 | - | 0.009 |
| Pyr-RSNP | NMDA | 0.02 | 0.005 | 0.150 | 0 | 0.020 | 0.015 |
| Basket-Pyr | GABA | 0.0 | 0.0 | 0.006 | -85 | - | 4.11 |
| Basket-Basket | GABA | 0.0 | 0.0 | 0.006 | -85 | - | 0.25 |
| RSNP | GABA | 0.0 | 0.0 | 0.006 | -85 | - | 2.2 |

Table A2. Table of synapse parameters. When a spike is received the conductance grows asymptotically to the maximum value, with characteristic time constant τ_raise_(if 0 instant rise to maximum value). Conductance decays asymptotically to 0 with characteristic time constant τ_decay_. Duration is the time interval from start of raise phase to start of decay phase. E_slow_ is the reversal potential of the NMDA calcium current.

| Particle/  Channel | $\alpha$A(V^-1^s^-1^) | | | $\alpha$B(V) | $\alpha$C(V) | $\beta$A(V^-1^s^-1^) | | | | $\beta$B(V) | | $\beta$C(V) |
| --- | --- | --- | --- | --- | --- | --- | --- | --- | --- | --- | --- | --- |
| Na activation | 0.58e6 | | -50e-3 | | 1e-3 | 0.174e6 | | | -59e-3 | | 20e-3 | |
| Na inactivation | | 0.232e6 | | -50e-3 | 1e-3 | 1.16e3 | -46e-3 | | | | 2e-3 | |
| K activation | 0.058e6 | | | -50e-3 | 0.8e-3 | 0.0145e6 | | -40e-3 | | | 0.4e-3 | |
| Ca activation | 0.232e6 | | | -10e-3 | 11e-3 | 0.0029e6 | | 10e-3 | | | 0.5e-3 | |
| NMDA | 2.03e3 | | | - | 17e-3 | 29.232 | | - | | | 17e-3 | |

Table A3. Table of channel particle dynamics.

**References**

1. Ekeberg Ö, Wallén P, Lansner A, Tråvén H, Brodin L, Grillner S (1991) A computer based model for realistic simulations of neural networks. Biol Cybern 65: 81-90.
2. Franzén E, Lansner A (1998) A model of cortical associative memory based on a horizontal of connected columns. Network: Comput Neural Systems 9:235-264.
